# The Impact of Pelvic Organ Prolapse on the Long-Term Outcomes of Transobturator Tape (TOT) Procedures—A Retrospective Study

**DOI:** 10.3390/jcm14010159

**Published:** 2024-12-30

**Authors:** Alessia Hillmeyer, Lieven N. Kennes, Mila Strauss, Katharina Lube, Elmar Stickeler, Laila Najjari

**Affiliations:** 1 Department of Obstetrics and Gynecology, University Hospital RWTH Aachen, 52074 Aachen, Germany; 2 Department of Econometrics and Statistics, University Stralsund, 18435 Stralsund, Germany

**Keywords:** TOT, SUI, mesh-erosion, mesh complications, revision rates

## Abstract

**Objectives**: Transobturator tape (TOT) procedures are a widely used and effective treatment for stress urinary incontinence (SUI), but there is limited research on mesh-related complications and revision surgeries. This study aimed to evaluate the incidence of revision surgeries and mesh-related complications following TOT procedures and identify potential risk factors influencing these outcomes. **Methods**: This retrospective study analyzed data from patients who underwent TOT procedures at the specialized incontinence center of University Hospital Aachen (UHA), Germany, between January 2010 and May 2023. Patients were divided into three groups: initial surgery without revision, initial surgery with revision, and external referrals requiring revision. Statistical analyses included multivariate logistic regression and predictive cross-validation to identify risk factors for revision and mesh-related complications. **Results**: Out of 265 TOT procedures performed, the revision rate was 8.7%, and the mesh-related complication rate was 2.6%. Mesh complications, including erosion and wound dehiscence, accounted for 30% of revisions, while 70% of revisions were caused by recurrent stress urinary incontinence (SUI). External referrals showed longer revision intervals compared to UHA patients (53 months vs. 5 months; *p* = 0.003). Multivariate analysis identified rectoceles as a protective factor against revisions (*p* = 0.0414), while pre-existing conditions significantly increased revision risk (*p* = 0.0100). **Conclusions**: The revision rate following TOT procedures was 8.7%, with mesh-related complications accounting for 2.6%. Pre-existing conditions significantly increased the risk of revision, while rectoceles were associated with improved outcomes. These findings emphasize the importance of identifying patient-specific risk factors to enhance the safety and success of TOT procedures.

## 1. Introduction

Urinary incontinence (UI) is a common condition among women, with reported prevalence worldwide ranging from 5% to 70%, depending on patients’ demographics [1]. UI prevalence is notably higher in older patients, affecting over 40% of women over 70 years old [1]. Due to the high occurrence of this diagnosis, the probability for women to undergo surgical intervention for incontinence in their lifetime is significant. A study by Wu et al. conducted between 2007 and 2011 found that women have a cumulative lifetime risk for a vaginal sling operation of 14% [2]. Midurethral slings (retropubic and transobturator tapes) are commonly used to treat stress urinary incontinence (SUI) due to good treatment outcomes [3].

Given the widespread use of synthetic materials in urogynecological procedures and the increase in sling operations over the past decade [4], it is essential to conduct a thorough analysis of associated risk factors and complications. Two primary mesh-related complications are mesh erosion and mesh exposure [5,6]. Additional complications such as bladder and urethral perforation, neurological symptoms, urge incontinence or voiding dysfunction, pain, and dyspareunia have also been associated with synthetic slings [5,6]. The full extent of long-term adverse effects following sling operations remains unclear, as there is currently no comprehensive database tracking potential complications and insufficient longitudinal studies [5,7]. Patients with mesh-related complications often require one or multiple revision surgeries to extract or revise the mesh material. The limited long-term studies that have been conducted found that 6–7% of women who initially underwent a sling operation required surgical revision, and 3–4% underwent a mesh extraction [8,9,10].

Midurethral sling operation techniques (retropubic vs. transobturator tapes) have been widely discussed in relation to complication and revision rates. According to the European Association of Urology and the ESTER systematic review, retropubic mid-urethral sling operations are associated with cure rates of 89.1%, while transobturator mid-urethral slings have a lower cure rate of 64.1% [11,12]. Although retropubic slings are associated with higher cure rates, they have a higher incidence of bladder perforations, bladder voiding dysfunctions, and retropubic pain or hematomas [13,14]. Conversely, transobturator slings are associated with a higher risk of groin pain and vaginal injuries [14,15]. While retropubic slings have a higher risk of intraoperative complications, transobturator approaches have a higher sling revision rate and lower subjective cure rates [15,16]. By analyzing the TOT complications of our specialized incontinence center, our study aimed to further understand the long-term outcomes of this widespread procedure.

As external factors significantly impact mesh-related complications, it is imperative to identify specific risk and protective factors for these complications. Studies have found that patients treated in specialized centers have a lower complication rate, as the treating surgeon’s experience has a significant effect on the patient’s outcome [10]. Additionally, postoperative ultrasound imaging is an important diagnostic tool for reducing mesh erosion, as incorrect sling placement is a significant risk factor for mesh-related complications [17]. The type of mesh material used in urogynecological surgeries may also influence complications, but the available data is still limited [18,19]. Some studies have shown that polyvinylidene difluoride (PVDF) materials cause less de novo urge incontinence and sexual dysfunction symptoms compared to the previously used polypropylene (PP) material [19]. Even though PVDF materials do not lower erosion rates or rates of recurrent SUI, they have been found to be a safe and effective alternative to PP materials [19,20]. Urogynecological comorbidities, such as anterior or posterior wall prolapse, are factors that may impact or complicate the treatment of SUI [21,22]. The full extent of this impact remains unknown and is one of the main research objectives of this paper.

This study aimed to evaluate the incidence of revision surgeries and mesh-related complications following TOT procedures and identify patient-specific factors that influence surgical outcomes, including pre-existing conditions and prolapse types. This information will be valuable in improving the quality of long-term care provided to patients undergoing urogynecological surgeries.

## 2. Materials and Methods

### 2.1. Study Design

This retrospective study used data from patients who underwent TOT surgery at the University Hospital Aachen (UHA) specialized incontinence center between January 2010 and May 2023.

### 2.2. Eligibility Criteria

The data were collected using the Department for Obstetrics and Gynecology surgical database. The study included all patients who underwent a transobturator tape (TOT) insertion to treat stress urinary incontinence, conducted by the same surgeon during the study period. The database search also included cases of internal revision surgeries and revisions of patients who had not been initially operated on at UHA. In cases where patients had multiple revision surgeries, only the first revision surgery was included in the statistical analysis. As this study aimed to assess the long-term effects, patients with incomplete files and insufficient follow-up data were excluded from the analysis. Based on the database search results, we defined three subgroups of patients: (A) those who had their initial surgery at UHA without subsequent revision, (B) those who had both their initial surgery and revision at UHA, and (C) those who only had revision surgery at UHA.

### 2.3. Preoperative Evaluation

The primary indication for surgical intervention was the diagnosis of stress urinary incontinence (SUI). The decision to pursue a surgical treatment was reached in agreement with the patient, considering the severity of ongoing SUI symptoms and after conservative treatments, such as local estrogen therapy and pelvic floor muscle training, had proven unsuccessful. Before undergoing the procedure, patients were thoroughly evaluated and diagnosed with SUI and any other urogynecological comorbidities. A specialized team conducted a physical examination, transvaginal ultrasounds, urodynamic investigations, a stress test, and calculated the International Consultation on Incontinence Questionnaire (ICIQ) Score.

### 2.4. Surgical Procedure

A specialized surgeon performed a standardized transobturator sling operation on all patients using polyvinylidene fluoride (PVDF) mesh material (DynaMesh^®^-SIS, P.J. Dahlhausen & Co. GmbH, Cologne, Germany). The sling was inserted by making a lengthwise incision below the external urethral ostium and then tunneling bilaterally toward the obturator foramen. At a 45° angle, the obturator foramen was perforated using a guiding tool until the tip emerged internally into the vagina. This was done bilaterally approximately 4 cm laterally of the clitoris through a small incision of 1 cm. The TOT tape was passed through the incisions, ensuring correct placement and tension-free positioning under the urethra. Following an intraoperative stress test, where pressure was applied above the pubic symphysis, the tape was adjusted if necessary. If simultaneous operations, such as laparoscopic sacrocolpopexy or cervicosacropexy and anterior or posterior colporrhaphies, were indicated, these were performed according to surgical standards.

### 2.5. Postoperative Evaluation

Postoperatively, all patients underwent a comprehensive assessment prior to discharge. This included a thorough gynecological examination and an inspection of the surgical site. Ultrasound was used to verify correct sling positioning and to perform a post-void residual (PVR) test to diagnose potential voiding dysfunction. Patients were advised to return for a six-month follow-up evaluation, which included a sonographic assessment to monitor for complications or recurrence of symptoms.

### 2.6. Variables

The variables assessed for the descriptive statistical analysis were categorized into patient demographics, comorbidities, and surgical variables. The patient demographics included age, weight, Body Mass Index (BMI), parity, ASA Score (American Society of Anesthesiologists Physical Status Classification System), and history of pre-existing conditions. Pre-surgery treatments, such as other (uro-)gynecological surgeries (hysterectomy, anterior and posterior colporrhaphy), behavioral therapy (pelvic floor muscle training or bladder training), drug therapy, and electrical stimulation, as well as the ICIQ Score (calculated from the International Consultation on Incontinence Modular Questionnaire-short form), were additional factors analyzed. Comorbidities such as cystocele (ICS Stage 0–IV), rectocele (ICS Stage 0–IV), uterine prolapse (ICS Stage 0–IV), urge, and mixed urinary incontinence were examined in relation to the occurrence of major complications. Surgical variables were defined as the execution of simultaneous laparoscopic procedures (laparoscopic sacrocolpopexy or cervicosacropexy), hysterectomies, and anterior or posterior colporrhaphies.

### 2.7. Statistical Analysis

The distribution of the variables named above, both overall and within the three subgroups (Groups A, B, and C), was examined. Continuous variables are presented as mean values ± standard deviation (SD), while categorical data are shown as absolute frequencies and/or percentages.

For group comparisons, Fisher’s exact test was used for categorical variables, and the Mann–Whitney U-test was employed for continuous variables. These variables are presented as median values with an interquartile range (IQR).

To identify the most influential variables associated with complications and to classify patients accordingly, we conducted a multivariate logistic regression analysis and a predictive leave-one-out cross-validation model for Groups A and B.

The multivariate logistic regression, which included a variety of variables (detailed in the Section 3 (Results section)), was performed to examine its impact on complication status (no complications vs. complications during surgery). The odds ratio (OR) and 95% confidence interval (CI) were also included in the results of the multivariante analysis. We also calculated the area under the receiving operating characteristics (AUROC) for the multivariate analysis. To assess the model’s prediction performance on unseen data, we employed the leave-one-out method: for each participant, a logistic regression model was trained using the remaining 290 subjects. The model was then used to predict the probability of complications for the left-out participant. Given the imbalanced data (242 subjects with no complications and 23 with complications), participants were classified as having complications if the predicted probability exceeded 15%; otherwise, they were classified as having no complications. This process was repeated for all 291 subjects. The relative frequency of correct classifications is referred to as the accuracy. Additionally, to address the data imbalance, we calculated the balanced accuracy, which is the mean of the true positive rate and the true negative rate. It is important to note that patients with complications referred from other hospitals (Group C) were not included in the multivariate analysis and only analyzed descriptively or directly compared to Group B.

A *p*-value of <0.05 was considered statistically significant. Given the exploratory nature of this study, the significance level was not adjusted for multiple comparisons. All statistical analyses were performed using R software (Version 4.3.2) [23].

## 3. Results

### 3.1. Study Population

**Figure 1:**
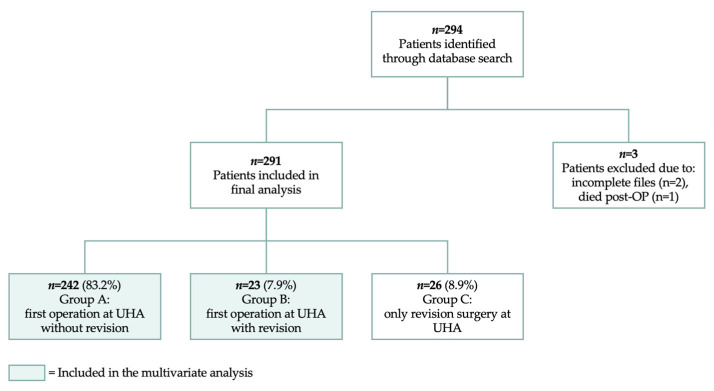
Patient flow diagram.

The internal database search identified 294 women who underwent a TOT operation at UHA during the study’s timeframe. Two patients were excluded due to incomplete files and insufficient follow-up data, and one patient died postoperatively due to a pre-existing cardiac condition. The remaining 291 patients were divided into three subgroups: (A) those who had their initial surgery at UHA without subsequent revision (*n* = 242), (B) those who had both their initial surgery and revision at UHA (*n* = 23), and (C) those who only had revision surgery at UHA (*n* = 26; see Figure 1)

### 3.2. Patient Characteristics

The study included participants with an average age of 59.50 years (SD = 13.04). The mean ICIQ Score of all participants was 15.18 (SD = 4.66), and 40.9% (*n* = 119) were diagnosed with SUI only. Before the surgical treatment in this study, 39.4% of women underwent behavioral therapy (pelvic floor muscle training), 16.8% underwent drug therapy, and 7.9% underwent electrical stimulation. Urogynecological comorbidities included cystoceles (38.1%), rectoceles (23.0%), uterine prolapse (17.2%), and simultaneous urge urinary incontinence (13.1%). Some participants had prior gynecological surgeries, with hysterectomies being the most common (43.0%), as well as non-gynecological surgeries (20.6%), such as open-heart surgery or oncological surgeries. Pre-existing conditions were observed in 40.2% of participants, resulting in a mean ASA score of 2.00 (SD = 0.64). The participants’ average weight at the time of operation was 75.07 kg (SD = 75.07), with a mean BMI index of 27.83 (SD = 5.74), classified as overweight. The study participants had an average of two pregnancies, with only 4.5% of women being nulligravida. 88.3% of participants had a vaginal delivery, while 12.3% had a cesarean section (see Table 1).

### 3.3. Revision Rate and Mesh-Related Complication Rate

Of the 265 patients who had their initial TOT insertion at UHA (Group A and Group B), 23 experienced complications requiring a subsequent revision (Group B). The revision surgery rate at this specialized center was 8.7%. Mesh-related complications occurred at a rate of 2.6%, including seven revision surgeries, three due to mesh erosion and four due to mesh-related wound dehiscence. Mesh-related complications accounted for 30.4% of UHA revisions. The remaining sixteen revision surgeries (69.6% of all UHA revisions) were performed to address recurrent SUI (*n* = 14) or acute postoperative sling-related voiding dysfunction (*n* = 2; see Table 2).

Revision surgeries for patients with mesh-related complications (*n* = 7) involved the excision of exposed mesh material, followed by a tension-free secondary wound closure. Patients with recurrent SUI (*n* = 14) were treated by inserting a second transobturator tape. In seven of these cases, the original tape was either partially (*n* = 2) or completely removed (*n* = 5) during the revision surgery. For patients experiencing acute postoperative voiding dysfunction (*n* = 2), the tape was loosened during the revision surgery to alleviate the symptoms. After the initial tape surgery, the postoperative ultrasound evaluation of all twenty-three patients in Group B had shown a correct TOT placement. Out of the fourteen patients who developed recurrent SUI symptoms, only three described mild SUI symptoms at the time of discharge after the first tape surgery.

Twenty-six patients were referred to our specialized center, needing revision surgery after undergoing a previous TOT operation elsewhere (Group C). Among these patients, 57.7% (*n* = 15) experienced mesh-related complications, including mesh erosion (*n* = 6) and mesh tension leading to urinary dysfunction (*n* = 9). The remaining 42.3% (*n* = 11) required revision surgeries due to recurrent SUI (see Table 2). Patients experiencing mesh erosion and tension were treated by removing the mesh material, while those with recurrent stress SUI underwent the insertion of a new TOT to alleviate their symptoms. During the preoperative ultrasound evaluation, patients with mesh erosions had atypical tape placements, which included intraurethral tape penetration, retrourethral erosions, or vaginal erosions. Additionally, patients suffering from urinary dysfunction related to mesh tension showed evidence of urethral compression during the preoperative ultrasound assessment.

The statistical analysis showed a significant difference in the time elapsed between the initial surgery and the revision surgery between the two groups (*p* = 0.0003). UHA patients had a median interval of 156 days, approximately five months, between surgeries. Externally referred patients had a median interval of 1620 days, more than ten times that of internal patients at around fifty-three months (see Table 2).

### 3.4. Risk Factor Analysis

Patients who initially received treatment at UHA and did not require further surgical revision showed a higher incidence of rectoceles (26.9%) and uterine prolapse (20.2%) compared to those who underwent revision surgery, with a prevalence of rectocele at 4.3% and uterine prolapse at 0%. These differences were found to be statistically significant (*p*(rectocele) = 0.020, *p*(uterine prolapse) = 0.011). Patients without revisions also demonstrated a higher incidence of cystoceles (41.3%) compared to patients with revisions (26.1%), although this difference was not statistically significant. Among participants who did not undergo revision surgery, 17.8% had a simultaneous laparoscopic procedure (laparoscopic sacrocolpopexy or cervicosacropexy) at the time of TOT insertion. This was statistically significant (*p* = 0.033) compared to the lack of simultaneous laparoscopies performed in the group that required revisions. The study results also revealed a significant difference (*p* = 0.012) in the prevalence of pre-existing conditions between both groups. The proportion of patients with comorbidities was substantially higher among those who had revision surgery, with 65.2% of these patients having at least one pre-existing condition. In contrast, only 36.2% of patients who did not have revision surgery had comorbidities. See Table 3 for the results of the risk factor analysis.

### 3.5. Multivariate Analysis

The variables included in the multivariate analysis were the presence of cystocele, rectocele, uterine prolapse, pre-existing conditions such as arterial hypertension, previous surgeries, simultaneous laparoscopic surgery (laparoscopic sacrocolpopexy and cervicosacropexy), and previous SUI treatment with electrical stimulation. The number of cesarean sections or abortions was also included. For the results of a univariate analysis of the variables, see Appendix A.

The multivariate analysis revealed that the variables rectocele (*p* = 0.0414) and pre-existing conditions (0.0100) had a statistically significant influence on the occurrence of complications and subsequent revision surgeries (see Table 4). The odds ratio for rectoceles (0.1751) indicates a possible protective effect against complications, suggesting that a lower presence of rectoceles is associated with an increased likelihood of complications. The odds ratio for pre-existing conditions (3.4095) indicates that patients with pre-existing conditions may have a higher likelihood of complications.

As detailed above, we calculated the accuracy and balanced accuracy using a leave-one-out method to assess the predictive power of our multivariate analysis. The accuracy is 0.8108, indicating that 4 out of 5 patients were correctly classified using the artificial intelligence-supported method. Additionally, to account for the imbalanced data, we calculated the balanced accuracy, which was 0.7588. This measure reflects the model’s ability to perform consistently well across both complication and non-complication cases, highlighting its robustness in handling imbalanced datasets. Additionally, the area under the receiver operating characteristic curve (AUROC) for the model was calculated to be 82.39%, indicating a strong discriminative ability to distinguish between the two groups. The graphical illustration of the ROC curve can be found in the Appendix B. These high accuracy and balanced accuracy rates underscore the strong predictive power of our multivariate analysis.

## 4. Discussion

Our research revealed an 8.7% overall revision rate and a 2.6% mesh-related complication rate for all TOT operations conducted at the specialized incontinence center of the University Hospital Aachen in over a decade. The need for revision surgery resulted from recurrent SUI (70% of cases) or mesh-related complications such as erosion and wound dehiscence (30% of cases). In comparison, a multi-center study in the State of New York, including over 35,000 women, reported a mesh erosion rate of 3.7% across all centers [9]. Other systematic reviews indicate a widely varying reoperation rate of up to 19%, based on the type of sling operation performed [24]. The high variability in recorded complication rates can be attributed to pre-operative comorbidities, patient characteristics (e.g., age, ethnicity), surgical procedures, surgeon experience, and poor data quality. Patients treated at a specialized center showed a lower risk for erosions compared to patients treated at hospitals where fewer midurethral sling operations were performed [9]. Our study further supports this evidence, as the 2.6% mesh erosion rate of the specialized incontinence center of the University Hospital in Aachen is lower than the mesh-erosion rates reported in multi-center studies [9].

Identifying potential risk factors is essential when assessing the long-term complication rates of TOT surgery. Our analysis revealed statistically significant differences in the characteristics of patients who initially underwent TOT treatment at UHA and later developed complications (Group A) and those who did not require revision surgery (Group B). UHA patients who required revision surgery showed a significantly lower incidence of rectoceles (4% vs. 27%; *p* = 0.020), uterine prolapse (0% vs. 20%; *p* = 0.011), and simultaneous laparoscopies (0% vs. 17.8%; *p* = 0.033). Our multivariate analysis indicated that the presence of a rectocele may be a protective factor against TOT revision surgery (*p* = 0.0414). This may seem surprising, especially as other pre-existing conditions (such as arterial hypertension, diabetes type II, cardiovascular disease, chronic obstructive pulmonary disease, etc.) were associated with a higher frequency of revision surgeries (*p* = 0.0100). However, studies have shown that posterior wall prolapse can significantly influence and even mask symptoms of stress urinary incontinence [25,26]. Although the exact mechanism behind this effect remains unclear, urodynamic evaluations have shown a higher urethral pressure in patients with posterior wall prolapse, potentially contributing to improved continence [26,27]. Furthermore, some studies have shown that the surgical correction of posterior wall prolapse can result in de novo SUI, a complication that is more commonly associated with anterior wall repair [28]. The findings discussed above do not fully explain the mechanisms behind our observation that rectoceles serve as a protective factor for TOT revisions. Still, they highlight a connection between the posterior compartment and incontinence that impacts the outcome of TOT surgeries and the need for revisions.

The retrospective design of our study allowed us to include almost 300 patients who underwent transobturator tape insertions at a single specialized incontinence center. Our single-center study design allowed us to minimize the impact of confounding bias on our results by having all operations performed under similar conditions by the same surgeon, using the same mesh material, operation technique, and pre- and postoperative evaluations. Our statistical multivariate analysis showed a high accuracy and balanced accuracy in assessing the relationship between TOT correction and possible preoperative risk factors, highlighting the high predictive power of our analysis and the robustness of our model in handling imbalanced datasets. Including data from patients referred to our center from other hospitals allowed us to have a more comprehensive view of patients who develop these rare complications.

Possible limitations of this study include the risk of selection bias, as some patients may have experienced complications and decided not to seek treatment or seek treatment elsewhere. However, the likelihood of patients being treated at another hospital for their TOT complication is comparatively low, as the incontinence center of the University Hospital is the primary referral center for urogynecological procedures in the area, and all patients underwent a 6-month follow-up exam. Furthermore, a single-center study design has its benefits in reducing confounding bias and achieving homogenous data, yet multi-center studies can recruit more patients and lead to better generalizability as the patient population is larger and more diverse.

Future research should include data on urodynamic evaluations and concomitant posterior colporrhaphies in their analysis. This could help to further investigate the impact of rectoceles on the outcome of TOT operations and explore the mechanisms behind why rectoceles were found to be a protective factor for revision surgeries. Finally, more long-term research on TOT complications should be conducted. Systematic reviews have tried to assess the long-term safety of midurethral sling procedures and often found incomplete data and short assessment periods of under 10 years [9,24]. Compiling a database to catalog these complications would be especially beneficial, as there is currently no systematic classification of long-term mesh-related complications.

## 5. Conclusions

This study demonstrated a revision rate of 8.7% and a mesh-related complication rate of 2.6% following transobturator tape (TOT) procedures performed at a specialized incontinence center. Pre-existing conditions were identified as significant risk factors for revisions, while rectoceles were found to be a protective factor, reducing the likelihood of complications. These findings emphasize the importance of comprehensive preoperative evaluation to identify patient-specific risk and protective factors that may influence surgical outcomes. The lower complication rates observed at a specialized center compared to the published literature underscore the value of surgical expertise and standardized procedures in achieving better outcomes. Future research should further investigate the mechanisms behind rectoceles’ protective role and focus on long-term data collection to improve the management of stress urinary incontinence.

## Figures and Tables

**Table 1:** Study population characteristics.

Study Population Characteristics	Results
Total participants (*n*)	291
Age in years (mean (SD ^1^))	59.50 (13.04)
SUI ^2^ as only diagnosis, *n* (%)	119 (40.9)
Urogynecological comorbidities, *n* (%):	
Cystocele	111 (38.1)
Rectocele	67 (23.0)
Uterine prolapse	50 (17.2)
Urge incontinence or overactive bladder	38 (13.1)
Prior surgeries, *n* (%):	
Hysterectomy	125 (43.0)
Anterior colporrhaphy	28 (9.6)
Posterior colporrhaphy	11 (3.8)
Vaginal sling operation	26 (8.9)
Sacrocolpopexy	14 (4.8)
Laparoscopic	10 (3.4)
Robotic assisted	4 (1.4)
Non-urogynecological surgeries	60 (20.6)
Simultaneous operations, *n* (%)	116 (39.9)
Pre-existing conditions, *n* (%)	117 (40.2)
ASA ^3^ Score (mean (SD ^1^))	2.00 (0.64)
Weight in kg (mean (SD ^1^))	75.07 (16.30)
BMI ^4^ in kg/m^2^ (mean (SD ^1^))	27.83 (5.74)
ICIQ ^5^ Score (mean (SD ^1^))	15.18 (4.66)
SUI ^2^ treatments prior to surgery, *n* (%)	
Behavioral therapy	115 (39.4)
Drug therapy	49 (16.8)
Surgical Treatment	56 (19.2)
Electrical stimulation	23 (7.9)
Parity (mean (SD ^1^))	2.16 (1.22)
Nulligravida, *n* (%)	13 (4.5)
Vaginal delivery, *n* (%)	257 (88.3)
Cesearian delivery, *n* (%)	37 (12.7)
Abortion, *n* (%)	18 (6.2)

^1^ Standard deviation. ^2^ Stress urinary incontinence. ^3^ American Association of Anesthesiologists Physical Status Classification System. ^4^ Body Mass Index. ^5^ International Consultation on Incontinence Questionnaire.

**Table 2:** Complication analysis: UHA complications and external complications (Group B vs. Group C).

	UHA ^1^ Complications (Group B)	External Complications (Group C)	*p*-Value
Total amount (*n)*	23	26	
Interval between initial operation and revision in days (median (IQR ^2^))	156 (1067)	1620 (2670)	0.0003
Type of complication, *n* (%)			0.001
Mesh related complications, *n* (%)	7 (30.4)	15 (57.7)	0.085
Mesh erosion	3 (13.0)	6 (23.1)	
Mesh-related wound dehiscence	4 (17.4)	0 (0.0)	
Mesh tension related urinary dysfunction	0 (0.0)	9 (34.6)	
Non-Mesh related complications, *n* (%)	16 (69.6)	11 (42.3)	
Recurrent SUI ^3^	12 (52.2)	11 (42.3)	
Recurrent SUI ^3^ due to sling dislocation	2 (8.7)	0 (0.0)	
Acute postoperative complications	2 (8.7)	0 (0.0)	

^1^ University Hospital Aachen. ^2^ Interquartile range. ^3^ Stress urinary incontinence.

**Table 3:** Statistically significant differences between UHA patients without revisions (Group A) and UHA patients with revisions (Group B).

	Initial Operation at UHA ^1^ Without Revision (Group A)	Initial Operation at UHA ^1^ with Revision (Group B)	*p*-Value
Total amount, *n*	242	23	
Cystocele, *n* (%)	100 (41.3)	7 (30.4%)	0.377
Rectocele, *n* (%)	65 (26.9)	1 (4.3%)	0.020
Uterine prolapse, *n* (%)	49 (20.2)	0 (0.0%)	0.011
Pre-existing conditions, *n* (%)	88 (36.4)	15 (65.2%)	0.012
Simultaneous laparoscopic surgery, *n* (%)	43 (17.8)	0 (0.0%)	0.033

^1^ University Hospital Aachen.

**Table 4:** Results of the multivariate analysis.

Variables	Estimate	OR ^1^	95% CI ^2^	*p*-Value
Arterial hypertension	0.4583	1.5814	[0.5705, 4.4024]	0.3349
Multiple previous surgeries	0.6584	1.9316	[0.6640, 5.7023]	0.1938
Pre-existing conditions	1.2266	3.4095	[1.3288, 10.5678]	0.0100
Cystocele	0.6384	1.8935	[0.6336, 5.5052]	0.2065
Rectocele	−1.7426	0.1751	[0.0059, 0.8413]	0.0414
Uterine prolapse	−1.9917	0.1365	[0.0000, 1.4844]	0.1424
Simultaneous laparoscopic surgery	−0.8214	0.4398	[0.0000, 5.9038]	0.5600
Previous electrical stimulation	−1.3856	0.2573	[0.0000, 2.2191]	0.3604
Cesearian delivery	1.0098	2.7451	[0.8527, 8.6783]	0.0642
Abortions	0.3856	1.4704	[0.1727, 6.0412]	0.6259

^1^ Odds ratio. ^2^ 95% confidence interval.

**Figure A1:**
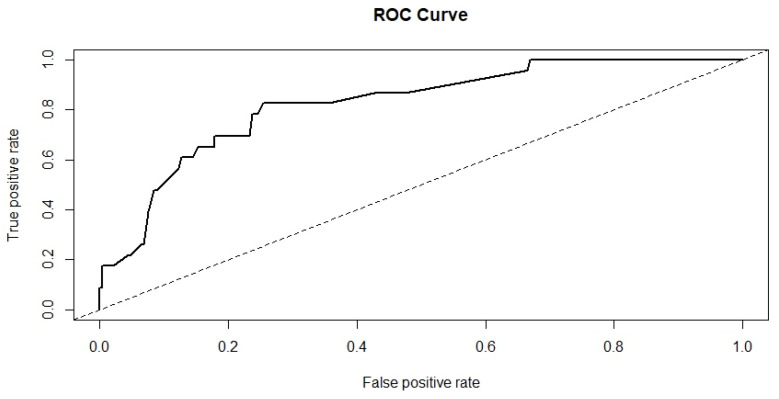
ROC ^1^ curve of the multivariate logistic regression model. ^1^ Receiver operating curve.

## Data Availability

Data available on request from the authors to protect patient privacy.

## Appendix A

**Table A1:** Results of the univariate analysis.

Variables	Estimate	OR ^1^	95% CI ^2^	*p*-Value
Arterial hypertension	0.2045	1.2270	[0.4843, 2.8730]	0.6429
Multiple previous surgeries	0.8094	2.2466	[0.8429, 5.3994]	0.0786
Pre-existing conditions	1.1728	3.2312	[1.3729, 8.5744]	0.0091
Cystocele	−0.4476	0.6391	[0.2294, 1.5246]	0.3322
Rectocele	−1.7029	0.1822	[0.0069, 0.7280]	0.0483
Uterine prolapse	−2.5183	0.0806	[0.0000, 0.5960]	0.0829
Simultaneous laparoscopic surgery	−2.3576	0.0946	[0.0000, 0.7025]	0.1052
Previous electrical stimulation	−1.4384	0.2373	[0.0000, 1.8311]	0.3329
Cesearian delivery	1.0819	2.9504	[0.9597, 7.6384]	0.0338
Abortions	0.5078	1.6617	[0.2122, 5.8825]	0.4908

^1^ Odds ratio. ^2^ 95% confidence interval.

## References

[1] Milsom I., Gyhagen M. (2019). The prevalence of urinary incontinence. Climacteric.

[2] Wu J.M., Matthews C.A., Conover M.M., Pate V., Jonsson Funk M. (2014). Lifetime risk of stress urinary incontinence or pelvic organ prolapse surgery. Obstet. Gynecol..

[3] Leone Roberti Maggiore U., Finazzi Agro E., Soligo M., Li Marzi V., Digesu A., Serati M. (2017). Long-term outcomes of TOT and TVT procedures for the treatment of female stress urinary incontinence: A systematic review and meta-analysis. Int. Urogynecol. J..

[4] Jonsson Funk M., Levin P.J., Wu J.M. (2012). Trends in the surgical management of stress urinary incontinence. Obstet. Gynecol..

[5] Brubaker L., Norton P.A., Albo M.E., Chai T.C., Dandreo K.J., Lloyd K.L., Lowder J.L., Sirls L.T., Lemack G.E., Arisco A.M. (2011). Adverse events over two years after retropubic or transobturator midurethral sling surgery: Findings from the Trial of Midurethral Slings (TOMUS) study. Am. J. Obstet. Gynecol..

[6] Bueno Garcia Reyes P., Hashim H. (2020). Mesh complications: Best practice in diagnosis and treatment. Ther. Adv. Urol..

[7] Ford A.A., Rogerson L., Cody J.D., Aluko P., Ogah J.A. (2017). Mid-urethral sling operations for stress urinary incontinence in women. Cochrane Database Syst. Rev..

[8] Gurol-Urganci I., Geary R.S., Mamza J.B., Duckett J., El-Hamamsy D., Dolan L., Tincello D.G., van der Meulen J. (2018). Long-term Rate of Mesh Sling Removal Following Midurethral Mesh Sling Insertion Among Women With Stress Urinary Incontinence. JAMA.

[9] Chughtai B., Mao J., Matheny M.E., Mauer E., Banerjee S., Sedrakyan A. (2021). Long-Term Safety with Sling Mesh Implants for Stress Incontinence. J. Urol..

[10] Welk B., Al-Hothi H., Winick-Ng J. (2015). Removal or Revision of Vaginal Mesh Used for the Treatment of Stress Urinary Incontinence. JAMA Surg..

[11] Nambiar A.K., Arlandis S., Bo K., Cobussen-Boekhorst H., Costantini E., de Heide M., Farag F., Groen J., Karavitakis M., Lapitan M.C. (2022). European Association of Urology Guidelines on the Diagnosis and Management of Female Non-neurogenic Lower Urinary Tract Symptoms. Part 1: Diagnostics, Overactive Bladder, Stress Urinary Incontinence, and Mixed Urinary Incontinence. Eur. Urol..

[12] Brazzelli M., Javanbakht M., Imamura M., Hudson J., Moloney E., Becker F., Wallace S., Omar M.I., Shimonovich M., MacLennan G. (2019). Surgical treatments for women with stress urinary incontinence: The ESTER systematic review and economic evaluation. Health Technol. Assess..

[13] Novara G., Galfano A., Boscolo-Berto R., Secco S., Cavalleri S., Ficarra V., Artibani W. (2008). Complication rates of tension-free midurethral slings in the treatment of female stress urinary incontinence: A systematic review and meta-analysis of randomized controlled trials comparing tension-free midurethral tapes to other surgical procedures and different devices. Eur. Urol..

[14] Thomas T.N., Siff L.N., Jelovsek J.E., Barber M. (2017). Surgical Pain After Transobturator and Retropubic Midurethral Sling Placement. Obstet. Gynecol..

[15] Tommaselli G.A., Di Carlo C., Formisano C., Fabozzi A., Nappi C. (2015). Medium-term and long-term outcomes following placement of midurethral slings for stress urinary incontinence: A systematic review and metaanalysis. Int. Urogynecol. J..

[16] Trabuco E.C., Carranza D., El Nashar S.A., Weaver A.L., McGree M.E., Elliott D.S., Linder B.J., Occhino J., Gebhart J.B., Klingele C.J. (2019). Reoperation for Urinary Incontinence After Retropubic and Transobturator Sling Procedures. Obstet. Gynecol..

[17] Viragh K.A., Cohen S.A., Raz S., Lo J., Raman S.S. (2018). Translabial Ultrasound in Midurethral Sling (Mesh) Visualization and Erosion Detection in Women with Stress Urinary Incontinence: A Retrospective Pilot Study. Ultrasound. Q..

[18] Sabadell J., Larrain F., Gracia-Perez-Bonfils A., Montero-Armengol A., Salicrú S., Gil-Moreno A., Poza J.L. (2016). Comparative study of polyvinylidene fluoride and polypropylene suburethral slings in the treatment of female stress urinary incontinence. J. Obstet. Gynaecol. Res..

[19] Karalis T., Tsiapakidou S., Grimbizis G.F., Mikos T. (2023). Surgical results in POP/UI surgery after using PVDF compared to other materials. A systematic review and meta-analysis. Eur. J. Obstet. Gynecol. Reprod. Biol..

[20] Barakat B., Hijazi S., Vögeli T.A. (2021). Use of polyvinylidene fluoride in treatment of female stress urinary incontinence: Efficacy and safety of midurethral slings: 24-month follow-up results. Turk. J. of. Urol..

[21] Wong J.W.H., Ramm O. (2021). Urinary Incontinence and Pelvic Organ Prolapse. Clin. Obstet. Gynecol..

[22] Pecchio S., Novara L., Sgro L.G., Rapetti G., Fuso L., Menato G., Biglia N. (2020). Concomitant stress urinary incontinence and pelvic organ prolapse surgery: Opportunity or overtreatment?. Eur. J. Obstet. Gynecol. Reprod. Biol..

[23] R Core Team (2023). R: A Language and Environment for Statistical Computing.

[24] Guillot-Tantay C., Van Kerrebroeck P., Chartier-Kastler E., Dechartres A., Tubach F. (2023). Long-term Safety of Synthetic Midurethral Sling Implantation for the Treatment of Stress Urinary Incontinence in Adult Women: A Systematic Review. Eur. Urol. Open Sci..

[25] Nguyen J.N., Yazdany T., Burchette R.J. (2007). Urodynamic evaluation of urethral competency in women with posterior vaginal support defects. Urology.

[26] Myers D.L., Lasala C.A., Hogan J.W., Rosenblatt P.L. (1998). The effect of posterior wall support defects on urodynamic indices in stress urinary incontinence. Obstet. Gynecol..

[27] Richardson D.A., Bent A.E., Ostergard D.R. (1983). The effect of uterovaginal prolapse on urethrovesical pressure dynamics. Am. J. Obstet. Gynecol..

[28] Smith T.M., DeLancey J.O., Fenner D.E. (2013). Post-reduction stress urinary incontinence rates in posterior versus anterior pelvic organ prolapse: A secondary analysis. Int. Urogynecol. J..

